# Could Alcohol Abuse and Dependence on Junk Foods Inducing Obesity and/or Illicit Drug Use Represent Danger to Liver in Young People with Altered Psychological/Relational Spheres or Emotional Problems?

**DOI:** 10.3390/ijms231810406

**Published:** 2022-09-08

**Authors:** Giovanni Tarantino, Mauro Cataldi, Vincenzo Citro

**Affiliations:** 1 Department of Clinical Medicine and Surgery, “Federico II” University Medical School of Naples, 80131 Naples, Italy; 2 Section of Pharmacology, Department of Neuroscience, Reproductive Sciences and Dentistry, “Federico II” University of Naples, 80138 Naples, Italy; 3 Department of General Medicine, “Umberto I” Hospital, 84014 Nocera Inferiore, Italy

**Keywords:** obesity, junk food, alcohol, illicit drugs, liver disease, young people

## Abstract

Recent data show that young people, mainly due to the pressure of some risk factors or due to disrupted interpersonal relationships, utilise greater reward value and display greater sensitivity to the reinforcing properties of “pleasurable stimuli”, specifically in those situations in which an enhanced dopamine release is present. Alcoholic beverages, foods rich in sugar and fat, and illicit drug use are pleasurable feelings associated with rewards. Research shows that there is a link between substance abuse and obesity in brain functioning. Still, alcohol excess is central in leading to obesity and obesity-related morbidities, such as hepatic steatosis, mainly when associated with illicit drug dependence and negative eating behaviours in young people. It is ascertained that long-term drinking causes mental damage, similarly to drug abuse, but also affects liver function. Indeed, beyond the pharmacokinetic interactions of alcohol with drugs, occurring in the liver due to the same metabolic enzymes, there are also pharmacodynamic interactions of both substances in the CNS. To complicate matters, an important noxious effect of junk foods consists of inducing obesity and obesity-related NAFLD. In this review, we focus on some key mechanisms underlying the impact of these addictions on the liver, as well as those on the CNS.

## 1. Introduction

Over the past 30 years in the USA, nonalcoholic fatty liver disease (NAFLD) has been the only liver disease with growing prevalence, synchronous with increasing rates of obesity and type 2 diabetes mellitus, and coupled with a nearly two-fold decrease in chronic hepatitis due to HCV infection, while the prevalence of HBV infection-related hepatitis and chronic alcoholic liver disease has remained stable [1]. Meanwhile, information concerning both historical and current prevalence, as well as mortality from national and international literature and databases on liver disease in 35 countries in the European region of the World Health Organisation, highlights alcohol consumption followed by obesity and hepatitis B and C virus infections as the main causes, confirming that the burden of liver disease in Europe continues to grow [2].

The prevalence of current alcohol use or binge drinking in adolescents aged 15 to 19 are 44 and 24% in Europe and 38 and 18% in the USA, respectively, with binge drinking defined as 60+ grams of pure alcohol (nearly four standard US drinks on at least one occasion/month), according to the World Health Organization’s 2018 global status report on alcohol and health [3]. Heavy episodic or “binge” drinking among college students is considered a major public health problem and has been reckoned as a principal cause of preventable death in this setting [4].

Drug use is widespread in every continent. For example, in the area of Southeast Asia there is an increasing trend towards drug utilisation among young people. In fact, a survey of students in northern Thailand showed that 30–40% of young males and 3–6 percent of young females have used cannabis, and that 18–20% of males and 12–27% of females have sniffed volatile solvents. The same survey showed that 5–10% of both males and females have used stimulants and nearly 2% have used heroin [5].

Adolescents and young adults are not specialised users of alcohol, tobacco, or marijuana, but rather tend to use or abuse multiple substances increasingly with age. Risk analyses have indicated that progression towards a substance-use disorder for any substance is increased with prior involvement with any of these three substances during adolescence [6]. The mental health of young men should be recognised as a major social issue leading to many problems such as unemployment, familial disruption and, mainly, substance abuse [7]. Adolescent mental health represents a neglected area of research and there is an unmet need to extend existing interventions as well as to devise new models to address this high-risk population [8]. Early intervention with these very young people can help prevent significant maladjustment and reduce their future need for mental health services. In fact, a systematic review was conducted that addressed the emotional and or mental health problems of university students worldwide, and ended up showing that the number of them with a serious mental illness has risen significantly over the past few years [9].

While the psychiatric consequences of illicit substance abuse have been extensively taken into account by various researchers and consequently largely advertised, less attention has been paid to the possible hepatotoxic effects. Illicit drug abuse may cause hepatic damage characterised by abnormal liver function tests, such as asymptomatic derangement, but also fulminant hepatic failure [10].

This interesting aspect will be addressed shortly considering overweight/obesity, eating, and physical activity patterns, even though several other factors play an important role in determining excess weight gain. In this context, junk foods and sugar drinks are found to be associated with obesity due to their high energy content and low nutritive value. The steep increase in obesity in every age group constitutes a major health problem worldwide. The prevalence of obesity in the USA has been found to be higher among adolescents aged 12–19 years (20.6%) than youths aged 6–11 years (18.4%) and children aged 2–5 years (13.9%) [11]. There is much debate around obesity as a disease state requiring treatment and prevention efforts or not [12], in the light of the fact that a subgroup of obese individuals has been described as metabolically healthy obese (MHO), which has also been found among adolescents [13]. The MHO phenotype is characterised by the absence of the criteria of metabolic syndrome in the presence of obesity. A recent piece of research challenges this definition. In fact, in one study, both MHO and metabolically unhealthy obesity groups, characterised by the same body weight, displayed significant impaired insulin sensitivity compared with the reference control [14]. The role of insulin sensitivity/resistance is central to obesity-related NAFLD, the main comorbidity of obesity. These observations lend credence to another key finding concerning the fact that about 20% of the MHO or metabolically healthy overweight at 13 years transition to metabolic unhealthy overweight/obese at 24 years [15].

Could risk factors, such as some aspects of adolescent behaviour (use of illicit drugs) and/or diet patterns concerning alcohol beverages and/or calorie-rich foods as well as fruit-flavoured drinks containing added sugar speed the transition from MHO to metabolic unhealthy obese, characterised by chronic inflammation, oxidative stress and insulin resistance?

## 2. The Reinforcing Properties of “Pleasurable Stimuli” among Young People

First of all, we should present the causes of the “intense urges and cravings” experienced by alcohol and drug abusers among adolescents/young people. They are multiple and very complex, and include: social circumstances, such as isolation and loneliness; poor quality of life due to problems with disrupted physical and mental health, low levels of education, youth unemployment and scarce job satisfaction, as well as minimal recreation and leisure time and the presence of pollution and/or noise in the environment; social stigma and discrimination during the school years; environmental/developmental risk factors, such as parental alcohol/drug use, family conflicts, poor parenting and maltreatment; and personality development, including parental attitudes and stimulation, peer relationships, learning experiences and hereditary predispositions [16,17].

Nowadays, alcohol is particularly obtainable. Consistent antecedent risk factors for starting to drink in adolescence are parental or peer approval, models for drinking and drug use, as well as adolescents’ own prior involvement in “delinquent” behaviour [18]. Additionally, the idea that drinking could be a pleasurable experience plays a role, due to the fact that alcohol is successfully promoted throughout society all over the world [19]. Recently, it has been concluded that significant numbers of both male and female undergraduate students are reported to exceed sensible weekly consumption guidelines [20]. Indeed, some of the behavioural pharmacological effects attributed to ethanol may be a result of the formation of brain acetaldehyde [21]. These enzymatic mechanisms of ethanol oxidation in the brain with special reference to the mesocorticolimbic system (involving a dopaminergic brain pathway) play an important role in ethanol reinforcement [22].

Variables associated with overeating and the consumption of high-calorie foods inducing overweight/obesity include stress, sensitivity to reward, compulsive behaviour and eating style. Stress has been assessed by researchers as one of the more influential environmental factors that likely contributes to developing overweight/obesity and associated comorbidities [23]. Specifically, when examining the prevalence and associated risk predictors of overweight/obesity and perceived stress using the eating behaviours and physical activity of 4609 adolescent students, aged 13–19 years, the highest percentage, i.e., 61.5%, was characterised by a moderate to extremely severe levels of stress, of which 28.2% were overweight/obese, only 2.7% had a very active lifestyle, and 30.5% had a sedentary lifestyle [24]. Recent data show that adolescents utilise greater reward value and display greater sensitivity to the reinforcing properties of “pleasurable stimuli”, specifically in those situations in which the enhanced release of dopamine reveals an existence peak [25]. Risk-taking expands from childhood to adolescence as a consequence of adaptation during puberty in the brain’s socio-emotional system, leading to increased reward seeking, mostly under the heavy influence of peer pressure, sustained by an exaggerated remodelling of the brain’s dopaminergic system [26]. Interestingly enough, a recent study has shown that the obese take more risks, as evaluated by a gambling task [27]. Indeed, neuronal plasticity might be modified towards both an augmented disposition to eating disorders such as anorexia, bulimia and obesity, as well as drug addiction (an issue that we will develop later), in the case of some circumstances in which visualising food and then eating it is linked to particularly susceptible subjects to exaggerate increases in reward circuitry activity [28]. This shift is, much more interestingly, detected in changes in dopamine and glucose metabolism during neuroimaging [29]. However, another piece of investigation shows that increased neural activation in obese subjects, which happens during reward processing, is likely to manifest even without food-related stimuli, consequently emphasising nonspecific dysfunctions in reward-related brain circuits of the same subjects [30].

Research shows that there is a link between substance abuse and obesity in genetics and brain functioning. In fact, it has been hypothesised that the polymorphism of the D2 dopamine receptor, which is less responsive to dopamine stimulation, leads to self-stimulatory behaviour that is expressed in drinking alcoholic beverages, abusing illicit drugs, or binging on foods [31]. Moreover, chronic low-grade inflammation, characteristic of obesity and mainly of visceral obesity, is hypothesised to affect brain function, giving way to addictive behaviours. The latter, in turn, create a self-perpetuating cycle that might impair the brain’s normal relationship with food, but also prompt drug, alcohol and gambling addiction [32]. In a mouse model, a high-fat diet not only induced obesity and provoked hyperlipidemia, but also led to depressive and anxiety-like behaviours via increased neuroinflammation and reduced brain-derived neurotrophic factor level in the hippocampus [33].

Among the health risks of junk foods, cerebral dysfunction occupies a central place. It is well-known that saturated fat along with trans fats are unhealthy fats [34]. Junk foods are characterised by so-called hyper-palatability, with alluring combinations of fat, sugar, carbohydrates, and sodium. They have large amounts of saturated fats and very often contain high amounts of industrially produced trans–fatty acids, as a result of the production process involving partially hydrogenated fat. Compressively, total fat in junk foods can range from 20.8% to 36% [35]. Interesting pieces of research show that metabolic processes such as burning fuels in mitochondria may alter some aspects of synaptic plasticity, and consequently have the potential to affect cognitive function. In fact, the authors, by using the antioxidant power of vitamin E, ascertained the role of oxidative stress as a mediator for decreased levels of brain-derived neurotrophic factor in synaptic plasticity and cognition caused by the consumption of high-saturated-fat diets in a way that jeopardises neuroplasticity and cognitive function, and worsens the outcome of brain insults [36]. Up-to-date results suggest that the frequent consumption of high levels of sugar-sweetened beverages by adolescents could have a serious impact on the neurocognitive functions affecting decision making and memory, potentially yielding them at risk for developing mental health disorders [37]. Children eating a diet high in junk food are more likely to show hyperactivity [38]. This point will be further explored later. Anyway, it is important to note here that some attempts have been made at establishing a causal link as to how a high-fat diet, such as eating junk foods, could give rise to cerebral dysfunction in adolescents, and whether effects on brain function are permanent or reversible [39]. It is also evident that, in urban adolescents, depression is associated with junk food [40]. Accordingly, a survey on 5473 students showed that symptoms of depression/anxiety due to life stress were significantly related to unhealthy eating behaviours, independently from gender, parental education level and economic status [41]. An important noxious effect of junk foods consists of inducing obesity and obesity-related NAFLD, an issue that will be addressed shortly.

To the latter effect, “alcoholic fatty liver” should be added as a fearful consequence of abusing alcoholic beverages, especially in the case of prolonged drinking [42]. Whether the exacerbation of alcoholic liver disease in overweight/obese patients is a result of a supplementary injury from NAFLD, characterised by visceral fat depots with an inflammatory profile [43], or vice versa, the metabolic alterations characterised by obesity exacerbate ethanol-induced liver injury, remains a subject of deeper investigation. Interestingly, Shütze et al. discovered a positive association in men and no association in women between beer consumption and waist circumference (WC) at baseline. Specifically, they found that male heavy beer drinkers (more than one litre/day) had a 17% greater gain in abdominal fat deposition than very light drinkers during eight and a half years of follow-up. Furthermore, an increase in visceral adiposity was significantly lower in beer-abstaining women than in very-light-drinking women [44]. Finally, other recent studies have shown that heavy drinking may be more of a risk factor for weight gain than light-to-moderate drinking [45]. The authors found that the alcohol dehydrogenase-1B (ADH1B) genotype (rs1229984) is a central factor in determining the body weight of alcoholic individuals. Quicker ethanol elimination, associated with the ADH1B*2 allele, leads to the less effective utilisation of ethanol as an energy source [46]. A study of 1604 alcoholic patients revealed that overweight (as determined by BMI ≥25 in women and ≥27 in men) is a risk factor for the progression of alcoholic liver disease towards more severe forms, i.e., alcoholic cirrhosis [47]. Emerging evidence suggests that adipose tissue, mainly the visceral one, has a regulatory function in metabolism and immunity through cytokines such as TNFα, IL-6, monocyte/macrophage chemo-attractant protein 1, and adipokines, which may regulate the insulin resistance and tissue inflammation that is part of so-called chronic low-grade inflammation [48]. Moreover, chronic ethanol exposure results in inflammation in adipose tissue in mice [49]. Thus, both the direct effects of alcohol on the metabolic and the innate immune activity of adipose tissue likely contribute to expand ethanol-induced liver injury.

Fast foods, meals eaten out, and fried foods, all of which are representative of energy-dense diets, conceivably might increase the risk of obesity, mainly among the young [50]. Data from large cross-sectional studies, as well as prospective cohort studies with long-lasting follow-up, show a direct association between major intakes of sugar-sweetened beverages and overweight and/or obesity in children, but also in adults [51]. Investigating the effect of fast-food-based hyperalimentation on liver enzymes and hepatic triglyceride content (HTGC), the authors found that hyperalimentation per se can induce a deep alanine amino transferase increase in less than one month and augmented HTGC, which are clear signs of NAFLD presence [52].

Still, studying the impact of obesity on drug metabolism and elimination in adults and children, researchers have evidenced that the clearance of cytochrome P450 (CYP) 3A4 substrates is lower in obese patients compared to normal-weight subjects. In contrast, CYP2E1, another member of the superfamily of CYP 450 enzymes which are involved in the biotransformation of drugs, xenobiotics and endogenous substances [53], has been shown to be higher in obese versus nonobese patients, and in those suffering from metabolic syndrome [54,55]. Additionally, in obese patients, trends indicating higher clearance values have been seen for drugs metabolised via CYP1A2, CYP2C9, CYP2C19 and CYP2D6 [56]. Coming back to the increased activity of CYP2E1 in the obese, ethanol also raises its induction, leading to further oxidative stress via the production of excessive reactive oxygen species [57]. Furthermore, this induction has also been found at the hepatic level in patients with NAFLD [58].

## 3. Alcohol Effects and Side-Effects

Individuals indulging in alcoholic beverages are prone to drug toxicity because alcohol induces fatty liver injury and subsequently the loss of liver function due to cirrhotic transformations that potentially alter drug metabolism [59]. Furthermore, alcohol abuse causes the depletion of glutathione, which functions as a hepatoprotective factor, making drinkers more susceptible to toxicity by drugs [60]. Indeed, beyond pharmacokinetic interactions, in which alcohol interferes with the metabolism of drugs generally occurring in the liver, where both alcohol and many medications are metabolised frequently by the same enzymes, there are pharmacodynamic interactions in which alcohol enhances the effects of some drugs, particularly in the CNS, such as the case of antidepressants, antihistamines, barbiturates, benzodiazepines and opioids [61].

The evident effects experienced after a couple of beverages consist of the sensations of being relaxed and less reticent. Indulging in some more drinks can make subjects become more talkative, and their speech might become in some way slurred. Carrying on drinking makes them lack coordination. Alcohol-use disorders are associated with depressive episodes, severe anxiety, insomnia, suicide, and the abuse of other drugs. Health problems, when alcohol is consumed in large quantities and for a protracted time, include heart diseases such as cardiomyopathy, hypertension, heart failure and stroke, liver cirrhosis, and various types of cancers, such as head and neck cancer, oesophageal cancer, breast cancer and liver cancer. Heavy drinking can also cause mild anterograde amnesias, temporary cognitive deficits, sleep problems and peripheral neuropathy, gastrointestinal diseases, decreased bone density and pancytopenia. Finally, alcohol abuse can cause foetal alcohol syndrome due to the excessive consumption of alcohol by the mother during pregnancy [62,63,64,65,66,67,68,69,70,71,72].

However, why does alcohol have such a noxious effect on the liver? The speed of absorption of ethanol from the stomach (about 20% from the small intestine) depends mainly on the fed or fasting state of the drinkers, the concentration of ethanol in the alcoholic beverages, drinking patterns (such as continuous drinking, frequent heavy drinking or episodic drinking), time of day and dosage form [73]. The gender difference in alcohol levels is due mainly to a smaller gastric metabolism in females (due to the significantly smaller activity of class III alcohol dehydrogenase (ADH)), rather than to differences in gastric emptying or in the hepatic oxidation of ethanol [74]. Alcohol is eliminated from the body by various mechanisms. The first enzymes involved are aldehyde dehydrogenase (ALDH), gastric and hepatic ADH, cytochrome P450 (CYP2E1), and catalase. Gene variations of these enzymes deeply impact not only on alcohol consumption, but also on alcohol-dependent organ injury. Furthermore, it is becoming clear that noncoding variants in both ADH and aldehyde ALDH genes may influence alcohol dependence [75]. Specifically, the hypoxia of hepatocytes, interaction between alcohol metabolism byproducts and other cell components (resulting in the formation of harmful compounds—the so-called adducts), and highly reactive oxygen species (ROS) that damage other cell organelles, changing their redox state by altering the ratio of NADH to NAD+, are the most threatening consequences of alcohol metabolism [76]. The clarification of the pharmacokinetics of ethanol is very important for estimating the effects of ethanol on biological events, as previously reported [77]. However, the quantity and frequency of alcohol consumption in liver injury when associated with comorbidities such as obesity consequently bear ramifications concerning both the extent of tissue damage and response to drugs [78]. This is because the association of oxidative stress with apoptosis due to the presence of both high alcohol concentration and hyperglycaemia, often present in obesity, gives place to greater liver injury [79]. Deepening these underlying mechanisms, a suggestive finding is that impaired growth hormone-mediated signalling is found in ethanol-exposed hepatocytes. This process is due to the divergent effects of ADH- and cytochrome P450 2E1 (CYP2E1)-mediated ethanol metabolism on the Janus kinase 2 (Jak2)/signal transducer and activator of transcription 5b pathway [80]. Now, considering that the highly conserved and potent JAK/STAT signalling pathway is required for normal homeostasis, and when dysregulated contributes to the development of obesity [81], the role of alcohol on worsening hepatic steatosis, previously present as a consequence of obesity, is of great significance.

Dealing with junk foods such as potato chips, candy and soft drinks, an intriguing mechanism linking alcohol abuse, junk food and hepatic steatosis has recently been proposed, focusing on fibroblast growth factor 21 (FGF21). This hepatokine regulates sugar intake and preferences for sweet foods via signalling through FGF21 receptors in the paraventricular nucleus of the hypothalamus and correlates to reduced dopamine neurotransmission within the nucleus accumbens [82]. Accordingly, up-to-date results show that FGF21 also overcomes alcohol consumption through the same population of neurones in the brain, demonstrating its therapeutic potential in nonhuman primate models of excessive alcohol consumption [83].

However, FGF21 directly regulates lipid metabolism and reduces hepatic lipid accumulation in an insulin-independent manner [84], which is a key finding of NAFLD. Furthermore, plasma FGF21 level is significantly related to intra-hepatic triglyceride content [85].

## 4. Consequences of the Association of Alcohol Abuse with Illicit Drugs and Smoking Habits

Now, we include a new addiction (nicotine) that is very frequent among young people: the habit of smoking. Adolescents’ alcohol consumption, tobacco and illicit drug use are related and, ultimately, increase the odds of using other substances [86]. Abstinence (non-use), experimentation, regular use (both recreational and compensatory for other problems), abuse and dependency mould possible stages of teenage experience with both alcohol and drugs. Usually, teens abuse drugs for many different reasons, such as social/recreational reasons, coping, testing drug effects for curiosity, peer pressure, stress, a desire to escape [87] and, mainly, due social anxiety, on which many studies on young subjects are focused [88].

As previously mentioned, recreational use, taken for enjoyment or leisure purposes rather than for medical reasons, and the abuse of drugs associated with alcohol consumption at high doses by young people is frequent, and can lead to grave consequences, including depression and personality disorders, among others, from which behaviour problems follow. In the 15–24 year age range, almost a half of deaths (including accidents, homicides and suicides) involve alcohol or drug abuse [89]. Drugs and alcohol also contribute to physical and sexual aggression, such as assault or rape among college students [90]. At this point, we should come back to the habit of smoking among the young and very young. The repeated and regular recreational use of tobacco and alcohol to try to attenuate stress can lead to a vicious circle and other psychiatric diseases such as anxiety and depression. In fact, some teenagers, regularly using tobacco or alcohol to compensate for anxiety, depression, or a lack of positive social skills, in turn develop further anxiety. However, one of the main problems experienced by teenagers is that tobacco and alcohol can be “gateway drugs” for other drugs (marijuana, cocaine, hallucinogens, inhalants and heroin). The combination of teenagers’ curiosity, risk-taking behaviour and social pressure make it very difficult refuse. Teenagers burdened from a family history of alcohol or drug abuse and a marked deficit of pro-social skills can advance from experimentation to patterns of constant abuse or worse dependency.

Tantalising hints that coexisting mental disorders, including antisocial disorders, mood disorders, and anxiety disorders, may at least partially explain the dependence of young people on alcohol [91]. Alcohol disorders, in general, follow rather than precede the onset of other psychiatric disorders [92]. Drinking excessively is more frequent in adolescents with problematic Internet use compared to those without a compulsive use of it [93].

## 5. The Main Role of Stress

Whether stress is primitive or secondary in alcohol abusers is still debatable. The connection between stress and alcohol consumption was discovered long ago. Stress has been found to increase anxiety, and in turn alcohol is consumed to try to alleviate this anxiety. Further observations have shown that, in alcoholics, the physiological responses to stress are perturbed. These stress-induced modifications are mediated by the hypothalamic–pituitary–adrenal axis [94]. Chronic alcohol consumption is associated with elevated basal glucocorticoid secretion [95]. Moreover, beyond effects on mental health, stress also acts on the liver. Authors have found that stress, through the activation of different pathways, can over-activate the main immune cells of the liver, the well-known Kupffer cells, which form the basis of immune tolerance [96]. The same authors discovered that the over-activation of Kupffer cells can lead to injury to the liver by triggering neutrophils and producing reactive oxygen species through sympathetic nerve stimulation, the alteration of hepatic blood flow, and the intestinal flow of bacterial lipopolysaccharides. However, there is also clinical evidence that stress is a risk factor for liver disease [97]. In fact, a large meta-analysis in the UK showed that subjects with higher scores on a general health questionnaire that measured psychological distress had a higher mortality from liver disease [98]. Major depressive disorder and generalised anxiety disorder are overrepresented in nonalcoholic steatohepatitis subjects and are associated with more advanced liver histological abnormalities [99].

## 6. Liver Damage by Some Illicit Drugs

The most frequently used illicit drugs and their general characteristics and side-effects are as follows [100,101,102,103,104]:

Depressants are drugs that decline the central nervous system. These drugs decrease the concentration of subjects using them and damage their ability to react. Alcohol beverages, opioids (eroin), barbiturates and gamma hydroxybutiyric acid are the most frequent.

Stimulants are, vice versa, drugs that stimulate the central nervous system. These drugs typically increase energy, heart rate and appetite. Some examples include methamphetamine (speed, ice, base), cocaine, dexamphetamine and nicotine, but also common beverages and food such as caffeine and chocolate.

Hallucinogens are drugs which typically alter how an individual perceives reality. The subjects change the way they see, hear, taste, smell or feel things. Consequently, users experience things that are not real. Some examples of hallucinogens include ketamine and LSD.

### 6.1. Cannabis

This drug is generally dealt as a dry herb, but also as a powdery resin, and recently has begun to be sold online. It is mixed with tobacco and is smoked in a roll-up cigarette. Normally, young substance abusers feel relaxed, light-hearted and talkative, but can also feel anxious, too suspicious, or forgetful. The stems and leaves of cannabis contain less substance than germs, and because of this domestic cultivation has caught on among consumers.

### 6.2. Ecstasy

Anecdotal evidence suggests that the powder form of ecstasy is becoming increasingly popular on campuses. Ecstasy is a typical club drug. The effect is an “energy buzz” with a capacity for intense activity, which is followed by a sensation of peace and tranquillity. The side-effects consist of users suffering from dry mouth, tachycardia, blurred vision, chills, sweating, or stiffening of the limbs and jaw.

### 6.3. Amphetamines

Young people generally utilise amphetamines at social gatherings, i.e., parties, pubs, and clubs, or during the course of study, mainly at exam times. These drugs make users feel full of energy and excitement. They are used by people to overcome the restrictions of particular diets because the drug suppresses appetite. Common side-effects include difficulty sleeping, low energy levels and mood swings.

### 6.4. Khat

Khat is a flowering evergreen shrub, the leaves of which are chewed. It has similar effects and side-effects to amphetamines.

### 6.5. Mephedrone

Mephedrone belongs to a group of drugs that are closely related to amphetamines. There is very little evidence about mephedrone and what long-term effects it has.

### 6.6. Gamma-Butirrolattone

Gamma-Butirrolattone (GBL) is a “rave drug”, most popular on college campuses. GBL has the same effects as gamma-Hydroxybutyrate (GHB), which is commonly known as ‘liquid ecstasy’. GHB is also a naturally occurring metabolite of the inhibitory neurotransmitter gamma-aminobutyric acid found in the brain. It produces feelings of euphoria, reduces inhibitions, increases sexual performance and causes sleepiness. Potentially serious consequences are present when this substance is taken with alcohol or other depressants or sedative drugs.

### 6.7. Heroin

Pure heroin is a white-coloured powder and is generally sniffed, but is often smoked. Darker heroin can be injected. The main effect is a rapid reduction in emotional pain, more so than physical pain, by the induction of warm and drowsy feelings, which allow users to forget the troubles of their life. It creates a state of physical dependence among its users. The most frequent side-effect consists of overdosing, because sold-on-the street heroin is mixed with other substances manufactured in home labs by people attempting to convert prescription painkillers. Overdose can lead to coma or even death. First-time users are usually suffering from serious illnesses, such as cancer, and it can take relatively long periods to become addicted. Interestingly, withdrawal symptoms bear a great resemblance to flu-like symptoms.

### 6.8. Cocaine and Crack

Cocaine is bought as a white powder. It is normally sniffed but can be prepared for injection. Crack comes in the form of ‘chunks’ and can be smoked and injected. Habitual users feel particularly confident and strong. Dangerous consequences for users are becoming strongly dependent on the drug and finding themselves running into crime and violence due to the high price of it.

### 6.9. Ketamine

Born as an anaesthetic drug, it presents as a white crystalline powder. Beyond being a potent painkiller, it deeply alters perception with terrifying hallucinations. The most common side-effects are body numbness, convulsions, abdominal pain, dysuria and bladder dysfunction. Large doses can lead to feelings of intense paranoia.

### 6.10. LSD

D-lysergic acid diethylamide (LSD) is well known as “acid”. It is a powerful mood-changing substance, whose effects (trip) last for as many hours. Generally small squares of paper with cartoon designs are swallowed. Its use is particularly dangerous because car accidents can occur when users are not in control of their faculties.

### 6.11. Volatile Solvents

These substances, inopportunely and wrongly used, include nail varnish removers, aerosols, butane gas, glues, petrol and dry cleaning fluid. Sniffing these volatile substances make users feel lightheaded, as if they are drunk. Some users might experience hallucinations. Some substances are directly toxic to the liver, kidney, or heart and some produce peripheral neuropathy or progressive brain degeneration. Vomiting is frequent. Used by younger teenagers, they can unpredictably kill even first-time users.

Coming back to liver damage, it has been shown that cocaine can induce liver injury by conversion to toxic metabolites resulting from cytochrome P450 metabolism. In experimental animal models, the modulation of P450 activity (by the means of inducers, inhibitors or alcohol) modifies the relative toxicity and pattern of injury from cocaine. Vice versa, in humans, it is still in doubt whether hepatic injury is mediated by a toxic metabolite of cocaine as opposed to the direct effects of other insults, such as hyperthermia, anoxia or hepatic ischemia. The clinical presentation of cocaine hepatototoxicity consists of acute hepatocellular necrosis. At the beginning of the symptomatology, serum liver enzymes such transaminases and lactic dehydrogenase levels are markedly elevated, and vice versa, alkaline phosphatase concentration is generally slightly increased. The homeostatic process is impaired with clear signs of hypocoagulability and the prothrombin time rapidly increases, mirroring disseminated intravascular coagulation. The serum total bilirubin starts augmenting from the first days of use. Dermatologic expressions of allergy, such erythema and pruritus, are absent. Serum autoantibodies such as ANA, AMA and SMA are not detectable. Interestingly, liver histology is characterised by centrolobular (zone 3) necrosis and fat deposits, features that bear resemblance to ischemic hepatitis or liver injury due to hyperthermia [105]. In self-limited cases, serum transaminases levels usually return to the normal range in approximately 7–14 days, assuring a rapid recovery [106]. Unfortunately, the features are not the same, and not necessarily associated with a good prognosis. In fact, authors have reported a case of fulminant hepatic failure and acute rhabdomyolysis resulting from cocaine use. Coagulative-type, perivenular and mid-zonal necrosis and periportal “micro-vesicular fatty change” were the predominant morphological features throughout all lobules of the liver, in contrast to periportal necrosis described in the only previous case report with biopsy [107].

Using the method of transmission electron microscopy, the ultrastructural findings in the liver of intravenous heroin addicts were characterised by the hyperplasia and hypertrophy of the smooth endoplasmic reticulum, the vesicular degeneration of hepatocytes occurring as a result of the increased synthesis of the enzymes of the smooth endoplasmic reticulum, and the presence of a continuous basal membrane accompanied by the transformation of sinusoids into capillaries, leading to the impaired microcirculation that can ultimately progress in cirrhosis [108].

To deepen their understanding of this aspect, readers can consult a vast range of literature confirming the hepatotoxicity of cocaine in animal models and in humans [109,110,111,112,113,114,115,116,117,118,119,120,121,122].

Hyperthermia is a severe complication associated with the recreational use of 3,4-methylenedioxymethamphetamine (MDMA/ecstasy). Ecstasy-related deaths seem to be due to heatstroke from overheating as consequence of the dehydration of the body [123]. Ecstasy is responsible for a relatively high number of cases of acute liver failure in young people [124]. Many data indicate that MDMA is a central factor in MDMA-mediated hepatotoxicity through interaction with the glutathione system. When dealing with MDMA intoxication, the treatment with an antioxidant such as n-acetyl-cysteine may counteract the potentially hepatotoxicity. However, sulforaphane (potent antioxidant, present in cruciferous vegetables, such as broccoli, brussels sprouts and cabbage) supplementation, in this case, should be cautionary because of the possible drug–drug interaction [125]. On the other hand, the substances’ use affects nutritional status and body composition through decreased food intake and nutrient absorption and altered metabolism, in addition to the dysregulation of hormones altering the mechanism of satiety and food intake, such as leptin and grelin [126]. Paradoxically, malnutrition is also associated with NAFLD. In fact, in young children, it is associated with signs of hepatic dysfunction such as steatosis and hypoalbuminemia, but its aetiology is unknown [127].

To complicate matters, not all illicit drugs are characterised by hepatotoxicity. Δ9-tetrahydrocannabinolic acid (Δ9-THCA), the nonpsychotropic precursor of Δ9-THC, is one of the most abundant cannabinoids presents in cannabis sativa. Δ9-THCA prevents TGFβ-induced pro-fibrotic gene expression in vitro. Furthermore, it attenuates liver inflammation and fibrogenesis in vivo, providing a rationale for additional studies on the use of this cannabinoid for the treatment of liver fibrosis and the management of NAFLD [128]. Indeed, the complex effects and impacts of cannabinoids concerning NAFLD pathogenesis are still unclear, even though one of the possible mechanisms responsible for the positive effect of *Cannabis* use on NAFLD may include the antagonistic action of cannabidiol and delta9-tetrahydrocannabivarin on cannabinoid receptor type 1, beyond their anti-inflammatory properties [129,130,131].

Moreover, it should be stressed that daily cannabis smoking is significantly associated with fibrosis progression during chronic hepatitis following HCV infection. Such patients, often suffering from concomitant NAFLD, should be advised to refrain from regular cannabis use [132].

## 7. Mechanisms Linking Drug Abuse Immune Function and Inflammation

A relationship has been found between addictive drugs used abusively, such as alcohol and illegal drugs such as opiates, cocaine and marijuana, and increased susceptibility to infection [133]. In fact, when dealing with viral chronic liver diseases, substance abuse and recreational drug use are highly prevalent among HCV-infected patients [134]. There is evidence that certain illicit drugs, such as opioids, activate microglial cells and astrocytes, which causes central neuro-inflammation. In fact, opioids bind the Toll-like receptor (TLR)-4 leading to the augmented expression of nuclear factor kappa-light-chain-enhancer of activated B cells and the release of pro-inflammatory cytokines [135].

Studying the extracted RNA from homogenates of zebrafish exposed to illicit drugs, several differentially expressed sequences are associated primarily with the immune system, including several major histocompatibility complex class I and interferon-induced proteins. Interleukin (IL)-1 beta was found to be down-regulated in this interesting piece of research [136]. Mostly, these abnormalities are considered a risk factor for depression. In fact, patients with depression demonstrated higher concentrations of IL-1 beta in the cerebrospinal fluid. A positive correlation between serum IL-1β and the severity of depression also was observed [137]. Indeed, exploring the behaviour of tumour necrosis factor (TNF)-alpha and IL-1alpha levels in lifetime marijuana users versus no users, authors found a decrease in TNF-alpha in marijuana users, and there was no association of drug use with IL-1alpha [138]. Surprisingly, current findings support the previous literature, which presents the inverse relationship between IL-6 and neurocognitive dysfunction in self-reported lifetime marijuana users [139]. A significant reduction in interleukin (IL)-2 and an increase in anti-inflammatory transforming growth factor (TGF)-beta1, together with a diminution in the number of total lymphocytes, CD4 and natural killer (NK) cells, were found in the MDMA–cannabis group, with intermediate alterations in the cannabis group [140]. Cocaine use has been associated with a significant lowering in mitogen-induced lymphocyte proliferation, decreased cytokine formation, the impairment of dendritic cells, loss of T-cell stimulation, and impaired antibody formation [141]. A significant decrease in peripheral lymphocytes coupled with a significant increase in serum IgG, IgA and IgM was noted in the drug addicts [142].

Concomitant cannabis plus cocaine consumption coexists with pro-inflammatory status due to increased circulating amounts of lipopolysaccharide [143]. Chronic morphine and cocaine intake causes dysbiosis, increased intestinal permeability and a probable neuroinflammation, which could explain symptoms such as tolerance, hyperalgesia, and deficit in reward behaviour [144].

## 8. Discussion and Conclusions

The Diagnostic and Statistical Manual of Mental Disorders (DSM), which is the official text on which diagnoses are based, clearly links substance-use disorders with mental health problems [145]. However, what are the most frequent and contextually dangerous types of dependence in adolescent or very young people, in the light of their impaired psychological and relational spheres, as well as emotional problems? We have to connect the following epidemiological data before trying to answer this question. As previously seen, the prevalence of obesity is increasing more and more in young people [11]. About 15% of high school students reported having ever used illicit or injection drugs (i.e., cocaine, inhalants, heroin, methamphetamines, hallucinogens or ecstasy) [146]. Alcohol is the drug of choice among youth, with 12% of 8th-graders, 22% of 10th-graders, and 29% of 12th-graders reporting heavy episodic drinking [147]. Studying adolescents’ relationships with parents, other relatives, and mainly with peers, is a very interesting field of research that was recently accurately reviewed [148]. Mental, emotional and behaviour disorders in young people are burdened by high healthcare costs [149].

Focusing on the more widespread dependence that is alcohol abuse, we should take into account that it is central to leading to obesity and hepatic steatosis, mainly when associated with illicit drug use and negative eating behaviours. All of these factors could represent serious risk factors for liver damage, as we will discuss below.

Firstly, several mechanisms are at the basis of alcohol-induced liver injury. Research in animal models has found that the chronic consumption of ethanol markedly reduces both the amount and the activity of numerous antioxidant enzymes, which exacerbates the oxidant capacity of liver cells [150]. Furthermore, the interplay between CYP2E1-dependent oxidative stress, mitochondrial injury, stellate cell activation and glutathione (GSH) homeostasis may contribute to the toxic action of ethanol on the liver. Surprisingly, in one study, cells expressing CYP2E1 had elevated GSH levels. Similarly, levels of catalase, alpha-, and microsomal glutathione transferase were also increased, suggesting that there is an adaptive up-regulation of these antioxidant genes [151]. The latter reactions end up with the generation of lipid peroxides, which themselves interact with proteins and with acetaldehyde to form bulkier adducts (e.g., malondialdehyde-acetaldehyde (MAA adducts) that are capable of generating an immune response [152]. Furthermore, when the generated ROS undergo secondary reactions with adducts and unsaturated lipids of the hepatocytes, oxidant stress is worsened further. Finally, because of the high “substrate specificity” of CYP2E1s, increased levels of this enzyme accelerate the conversion of excess amounts of substrates other than ethanol, such as analgesics [153].

Secondly, authors in various pieces of research have concluded that liver fat excess could cause severe drug–drug interactions in patients with obesity-related NAFLD or nonalcoholic steatohepatitis. These interactions are related to the CYP2B6 and CYP2C9 enzymes [154,155,156]. The generation of ROS inside and outside of hepatocytes, the formation of reactive metabolites at hepatic level, the modification of covalent bonds between constituents of cells with drugs and their metabolites, the activation of signal transduction pathways that alter necrosis or apoptosis or survival pathways, characterised by wide-ranging changes in gene expression (i.e., impacting on the regulation of transcriptional and post-transcriptional activities) coupled with mitochondrial damage, which ends up in altering ATP generation, are all principal mechanisms of drug-induced hepatotoxicity [157].

Interestingly, we consider the reasons that drug addiction and the eating habits of patients suffering from obesity are characterised by same properties. Both are disorders in which a specific type of reward becomes exaggerated at the expense of other rewards. This mechanism is mediated by abrupt dopamine increases in the brain reward centre [158]. Indeed, sugar, the main constituent of some energy-dense, nutrient-poor foods, is highly palatable and rewarding, both in its taste and nutritive input, but excessive consumption can trigger neuro-adaptations in the reward system that decouple eating behaviour from caloric needs and lead to compulsive overeating. Excessive sugar intake is in turn associated with obesity [159] and obesity-related morbidities.

Both obesity and NAFLD are recognised as diseases characterised by gut flora dysbiosis [160]. Gut bacterial dysbiosis has long been observed in human alcoholic subjects, due to intestinal bacterial overgrowth and following gut leakiness [161].

Surprisingly, changes in the gut microbiome and its metabolites might not only be a consequence of substance-use disorders, but possibly play a role in mediating behavioural responses to drugs of abuse [162].

That said, a central point to be considered is the fact that there is an increased risk of developing a substance-use disorder in individuals with a mental health disorder relative to those without [163].

Conclusively, the question as to whether the use of illicit drugs and/or diet patterns comprehending alcohol beverages and/or calorie-rich foods could induce obesity should be rephrased as to whether these risk factors could impact, also and perhaps mainly, on liver function. The answer is positive and the potential summary of the aforementioned addictions is very dangerous for young people.

## 9. Future Directions

While dopamine release in the nucleus accumbent during social behaviours has been a topic of interest for the last three decades, many questions remain unaddressed. That dopamine does play a role in addiction has been ascertained, but likely some other factors—biological and environmental—are determinant to increase the risk in young people. Recent results support the hypothesis that nectin3 is a potential mediator of the effects of adolescent chronic stress on prefrontal structural and functional abnormalities [164].

An intriguing field of research linking a childhood mental health disorder such as attention deficit hyperactivity disorder (ADHD) to alcohol and other drug-related problems has given evidence that the persistence of this disorder into adolescence and adulthood makes subjects prone to addiction [165]. Surprisingly, dexamphetamine, which is indicated for the treatment of ADHD, shows direct and indirect evidence to support a potential therapeutical role in NAFLD, promoting satiety and reducing feeding through the activation of postsynaptic α- and β-adrenergic receptors and the D1/D2 receptor [166].

It is ascertained that long-term drinking causes mental damage, similarly to drug abuse. Among the social consequences of addiction, beyond the disruption of relationships, violence occupies one of the most important places. In fact, being drunk leads the young to become more prone to violence, theft and assault [62]. Additionally, not only young women, but also boys are affected by sexual violence.

However, is there a light at the end of the tunnel? Rational emotive behaviour therapy could help addicts recognise their negative thoughts and give them ways to fight self-defeating thoughts by reducing distress in high school students [167]. Contingency management, based on something of monetary value to incentivise addicts to not use drugs, is a highly effective treatment for substance use and related disorders [168]. Cognitive behavioural therapy is a valuable treatment tool because it can be used for many different types of addiction including, but not limited to, food addiction, alcohol addiction, and prescription drug addiction. In this sense, the role of psychotherapy in managing addiction is central. In fact, recent studies focus on using the process of forgiveness as a positive psychotherapy; whether this is implemented through stand-alone forgiveness interventions, infusion with twelve-step facilitation therapy, or application through acceptance-based treatment modalities remains to be established [169]. Researchers feel that making up a safe and nonjudgmental therapeutic alliance give patients the opportunity to increase their inner motivation to modify their attitude. Once motivation has been reinforced, a progressive decrease in substance intake can be obtained step-by-step, attenuating the neurobiological modifications caused by genetic predisposition and repeated substance use [170].

A diverse line-up of scientists think differently, pointing out new drugs. Accordingly, lofexidine is proposed to help reduce cravings and withdrawal symptoms in patients receiving treatment for opioid addiction [171]. Medications such as acamprosate can be utilised to reduce drinking behaviour [172].

To complicate matters further, genetic traits could impact addiction. In fact, in sample sizes of up to 1.2 million individuals, authors discovered 566 genetic variants in 406 loci associated with multiple stages of tobacco use (initiation, cessation, and heaviness) as well as alcohol use, with 150 loci evidencing pleiotropic association [173].

Much work has been completed and continues to be directed at developing reliable and practical means of the assessment of the link between diet components (so-called empty calories) and obesity in youth, the prevalence of which is increasing all over the word. However, many efforts should be directed at investigating the noxious effects of alcohol drinking on liver function among adolescents/young people, and of drug abuse at the same age. A determinant role is due to research in experimental animals. In fact, some authors feel that animal models of voluntary drug intake and addiction offer precious pieces of information for the identification of underlying mechanisms, and hopefully of drug development [174]. Finally, to gain further insight into therapeutical approaches to addiction and chronic metabolic dysfunction, such as obesity, we hope for clinical trials investigating new drugs and ultimately reaching satisfactory effects.
